# Mimicry of Rhabdomyosarcoma by Tonsillar Actinomycosis: Case Report

**DOI:** 10.3390/medicina60071172

**Published:** 2024-07-19

**Authors:** John Fernando Montenegro Palacios, Shirley Vanessa Correa Forero, Gaby Alejandra Ordoñez Andrade, Jasbleidy Posu Barco, Luis Alvaro Melo Burbano, Yamil Liscano

**Affiliations:** 1 Specialization in Internal Medicine, Department of Health, Universidad Santiago de Cali, Cali 5183000, Colombia; investigacion@clinicadeoccidente.com (S.V.C.F.); luis.melo03@usc.edu.co (L.A.M.B.); 2 Department of Research and Education, Clínica de Occidente S.A., Cali 760046, Colombia; barcojasble@gmail.com; 3 Genetics, Physiology, and Metabolism Research Group (GEFIME), Ciencias de la Salud Universidad Santiago de Cali, Cali 5183000, Colombia; 4 Ciencias de la Salud, Facultad de Medicina, Technological University of Pereira, Pereira 3137300, Colombia; aleja050799@gmail.com; 5 Grupo de Investigación en Salud Integral (GISI), Departamento Facultad de Salud, Universidad Santiago de Cali, Cali 5183000, Colombia

**Keywords:** actinomycosis, *Actinomyces europaeus*, *Actinomyces graevenitzii*, Gram-positive anaerobic bacteria, rhabdomyosarcoma, Positron Emission Tomography (PET-CT)

## Abstract

Actinomycosis is a rare infectious disease characterized by slowly progressive, chronic suppurative lesions, often mistaken for malignancies due to its ability to mimic them. It is caused by Actinomyces bacteria, which are part of the normal flora of the human oropharynx, gastrointestinal, and urogenital tracts. This case report describes a 51-year-old male with a history of mandibular rhabdomyosarcoma presenting with severe shoulder and hip pain, dysphagia, and headaches, initially suspected to be a cancer recurrence. However, after further investigation, including a PET-CT and tonsillectomy, the diagnosis of actinomycosis was confirmed through histopathological examination. The case highlights the diagnostic challenges of actinomycosis, especially in patients with complex clinical histories, emphasizing the importance of considering it as a differential diagnosis in similar presentations. The patient was treated with long-term antibiotic therapy, predominantly beta-lactams, demonstrating the necessity of a comprehensive diagnostic approach and the implications of a delayed diagnosis. This case underscores the critical need for high clinical suspicion and awareness among healthcare professionals regarding the potential for actinomycosis to mimic more common diseases, ensuring timely and accurate treatment.

## 1. Introduction

Actinomycosis is a rare infectious disease that progresses slowly and is characterized by the presence of chronic suppurative lesions. It is caused by anaerobic or microaerophilic bacteria, mainly from the genus *Actinomyces*. These bacteria belong to the *Actinomycetaceae* family and are identified as Gram-positive filamentous anaerobes [1]. This genus has over 30 species, some of which are part of the normal microbiota of the human oropharynx, gastrointestinal tract, and urogenital tract [1,2]. Although actinomycosis can occur in individuals of any age, it shows a higher incidence in men aged 20–60 years, with a peak incidence observed in those aged 40–50 years. Thus, although the condition is less common in very young individuals aged < 10 and adults aged > 60 years, it is not exclusive to any age group. Disease progression and its effect can vary, requiring a comprehensive approach to diagnosis and treatment [3,4].

*Actinomyces* spp. can mimic malignant tumors and infections such as those caused by *Nocardia* spp.; in Gram staining, this microorganism is indistinguishable from *Actinomyces* but can be detected in acid-fast staining.

It grows slowly, requiring incubation for at least 14–21 days for proper detection. The clinical presentation of actinomycosis can be challenging because it often mimics neoplasms, representing a significant diagnostic challenge. Specifically, tonsillar actinomycosis can mistakenly mimic conditions such as malignancies and tuberculosis, requiring a high level of clinical suspicion for an early diagnosis [1,4,5,6].

This report presents a clinical case of a patient with a history of mandibular rhabdomyosarcoma, in which findings suggestive of small cell sarcoma were identified in a biopsy of the proximal humerus. A positron-emission tomography-computed tomography (PET-CT) scan revealed hypermetabolic nodular thickening of the right lateral pharyngeal wall, suspicious of neoplasia. This case highlights the importance of considering rare chronic suppurative granulomatous infections [7,8].

Although rare, head and neck actinomycosis is a significant clinical condition owing to its varied presentation, which can mimic more common diseases, and the diagnostic and therapeutic challenges it poses [9]. Actinomycosis in the tonsils has an incidence rate of 1.3%. An increase in incidence has been reported in patients with underlying pulmonary disorders, which can further complicate the diagnosis if coexisting with diseases such as tuberculosis or cancer [10,11,12].

*Actinomyces europaeus* and *Actinomyces graevenitzii* show resistance to ceftriaxone [3,11,13]. Furthermore, these bacteria, normally present in the tonsillar crypts and able to proliferate on or directly invade tonsillar tissue, can have significant clinical effects, particularly in cases where the initial infection may mimic rhabdomyosarcomas [14]. This case underscores the importance of an exploratory approach in diagnosing complex pathological conditions.

## 2. Case Report

A 51-year-old male patient with a history of mandibular rhabdomyosarcoma presented with metastases to the left shoulder and right hip. Twenty-five years ago, he underwent parotidectomy for rhabdomyosarcoma and received specific oncological treatment with chemotherapy and radiotherapy, and he had been in remission since then. No other relevant medical history or subsequent clinical and paraclinical follow-up data were reported.

In 2022, the patient experienced worsening left shoulder pain with an intensity of 8/10 and right hip pain, which affected his mobility, thus preventing him from performing his daily living activities. In addition, he experienced dysphagia for liquids and solids, difficulty swallowing, and intermittent sharp parieto-occipital headaches. Occasional treatment with acetaminophen 500 mg every 12 h only provided partial relief. Physical examination revealed limited mobility in the left shoulder, restricted range of motion, and signs of instability in right hip mobility, associated with joint clicking, without apparent neurological deficit.

Given his oncological history of rhabdomyosarcoma with metastases to the left shoulder and right hip, he was evaluated by the orthopedic oncology service. Because of exacerbated pain, a biopsy of the proximal left humerus was requested in June 2022. Biopsy findings revealed a population of small morphologic tumor cells with hyperchromatism and scant cytoplasm, suggesting the presence of a small-cell sarcoma. Immunohistochemistry with hematoxylin and eosin staining revealed a scant representation of small blue tumor cells, which were not observed in the immunoperoxidase sections and, therefore, could not be evaluated.

**Figure 1:**
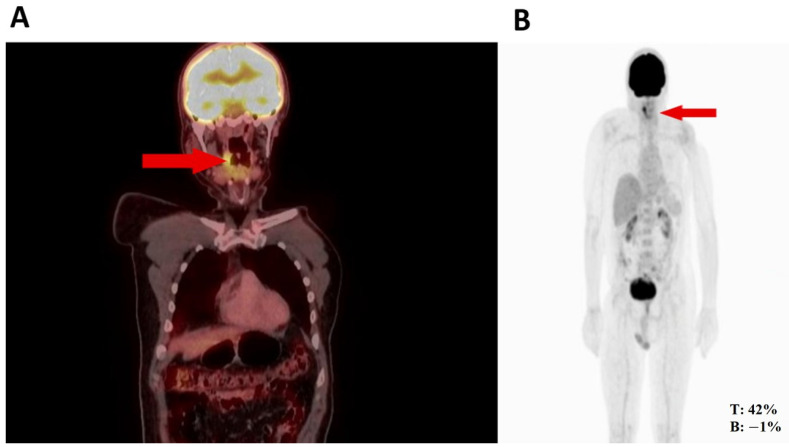
(**A**) Nodule in the right pharynx and palatoglossal arch measuring 22 × 10 mm, SUV 6.5 (red arrow). (**B**) Hypermetabolic nodular thickening of the right lateral pharyngeal wall, nonspecific, suspicious of neoplastic involvement (red arrows).

Thus, the orthopedic oncology service requested a PET-CT in August 2022, given its utility for diagnosing high- or low-grade sarcomas. Subsequently, the PET-CT (Figure 1) revealed hypermetabolic nodular thickening on the right lateral pharyngeal wall, which was suspicious of neoplasia. Based on the biopsy and PET-CT findings and history of rhabdomyosarcoma, an evaluation by the head and neck surgery service was indicated for surgical assessment.

**Figure 2:**
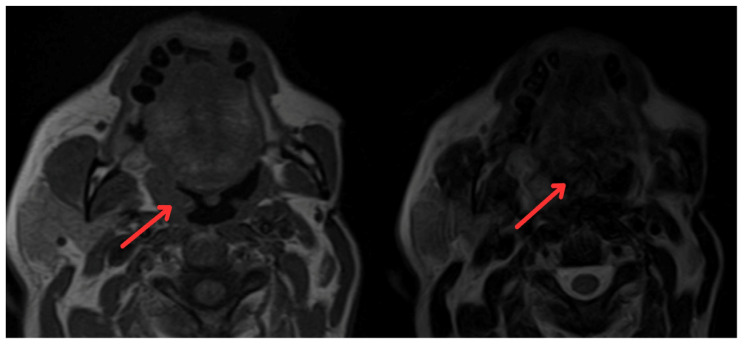
Magnetic resonance imaging of paranasal sinuses showing irregular enhancement forming a mass that involves the mucopharyngeal space and the right palatine tonsil (red arrows).

**Figure 3:**
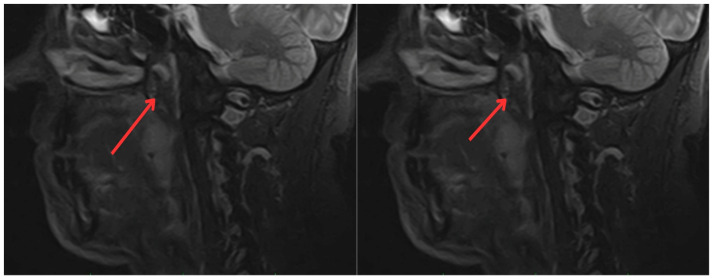
Contrast-enhanced magnetic resonance imaging of the neck: irregular enhancement, forming a mass that involves the mucopharyngeal space and the right palatine tonsil, extending to the tongue base, with a neoplastic appearance (red arrows).

In June 2022, the head and neck surgery service, suspecting a relapse of rhabdomyosarcoma, requested nasopharyngoscopy and magnetic resonance imaging (MRI) of the paranasal sinuses (Figure 2). The reports indicated the presence of a mass with irregular enhancement affecting the mucopharyngeal space and the right palatine tonsil and extending to the tongue base with neoplastic characteristics. This led to an expanded imaging study with a contrast-enhanced neck MRI (Figure 3), which showed irregular enhancement of a mass compromising the mucopharyngeal space and the right palatoglossal arch, measuring 22 × 10 mm, similarly affecting the right palatine tonsil and extending to the tongue base with a neoplastic appearance, as well as lymphadenopathy at the right cervical station three, which was suspicious.

Given these findings, a right tonsillectomy was considered in June 2023, and a biopsy + immunohistochemistry was attempted to achieve a more objective diagnosis.

**Figure 4:**
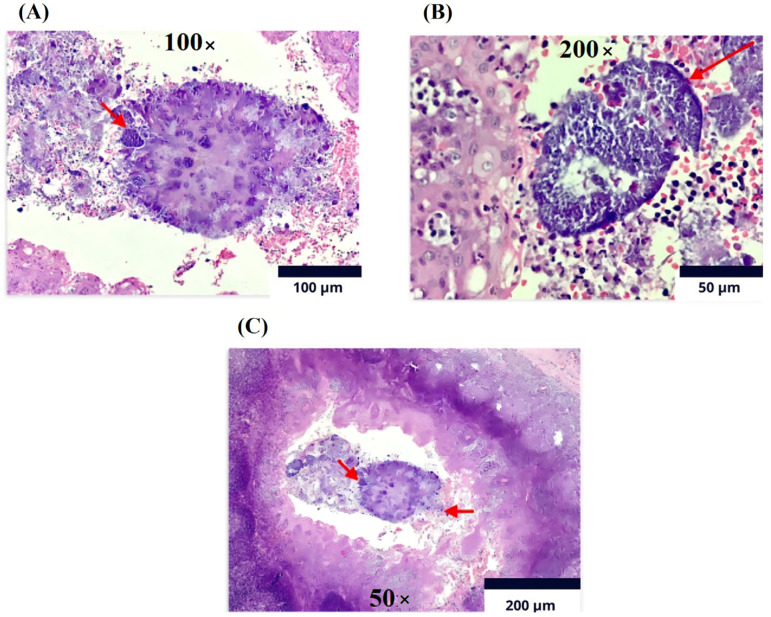
Immunohistochemistry (Olympus CX33 microscope). Lymphoid tissue with follicles of various sizes and shapes at the level of the cortex. (**A**). Magnification 100×: Reactive germinal centers with marked phagocytosis and Actinomycosis filamentary bacteria on the surface of sulfur granules (red arrow). (**B**). Magnification of 200×: The germinal centers are surrounded by a mantle of mature lymphocytes cured in sulfur granules due to actinomycosis (red arrow). (**C**). Magnification of 50×: In the crypts, detritus and clusters of polymorphonuclear cells and colonies of filamentous bacteria are present, compatible with *Actinomyces* spp. As observed, the arrow on the left indicates the polymorphonuclear reaction and the arrow on the right shows colonies of filamentous bacteria.

The histopathological report of the palatine tonsils revealed reactive follicular hyperplasia with evidence of filamentous bacterial colonies compatible with actinomyces (Figure 4), leading to a referral for evaluation by the infectious diseases service.

The infectious diseases service reevaluated the patient’s symptoms. In addition, the presence of fever, respiratory, urinary, and neurological symptoms was assessed, all of which were negative. The patient also denied any trauma or dental procedures. No solid nodules in the lung parenchyma nor mediastinal lymphadenopathy were found in previous thoracic CT images (July 2022), which allowed for the exclusion of pulmonary extension of the infection. Accordingly, the infectious disease service referred the patient to the emergency service for the induction of antibiotic therapy and to perform a brain MRI to rule out infectious involvement of the central nervous system.

The patient was hospitalized in July 2023, and blood cultures for 14-day cultures were taken, which were negative, and laboratory tests showed no abnormal values (Table 1 and Table 2). Unfortunately, as the initial diagnostic impression of the mass was a neoplastic condition, mass cultures were not taken during the surgical procedure. No pathological findings were noted on the brain MRI. Given the ease of administration once a day and considering an early hospital discharge, ceftriaxone 2 g every 24 h for 4 weeks was indicated as induction therapy through a peripheral venous catheter, followed by consolidation therapy with amoxicillin 2 g every 12 h for 12 months.

In the second week of treatment, the patient experienced slight bleeding from the peripheral venous catheter. After clinical evaluation of the catheters, thrombosis, active bleeding, or signs of infection were ruled out, allowing for hospital discharge. The patient completed the induction phase without complications and continued with amoxicillin 2 g every 12 h. He is now in his eighth month of treatment, with adequate clinical progress; no alterations were found on renal and liver function tests, and dysphagia was resolved.

## 3. Discussion

This report highlights the uniqueness and diagnostic challenges posed by actinomycosis, a rare infectious disease that progresses slowly and is characterized by chronic suppurative lesions. In this case, it was initially misdiagnosed as a tumor recurrence in a patient with a history of mandibular rhabdomyosarcoma. This case underscores the critical need to consider unusual differential diagnoses in patients presenting with complex clinical pictures, particularly when symptoms mimic more common diseases such as malignant tumors or tuberculosis [3,6]. A significant strength in this case was the exhaustive application of advanced diagnostic techniques, including biopsies, immunohistochemistry, and PET-CT. These methods initially pointed toward a neoplastic disease but eventually helped detect *Actinomyces* spp. However, a limitation was the late consideration of chronic suppurative granulomatous infections in the differential diagnosis. This delay highlights the importance of maintaining a high clinical suspicion for atypical infections in complicated clinical contexts.

Documented cases, such as those presented by Takasaki et al., 2006, Rašić et al., 2010, Samant et al., 2008, Yadav et al., 2002, and Bulut et al., 2014 [16,17,18,19,20], illustrate the variability in the clinical presentation of actinomycosis, ranging from significant enlargement of the tonsils to atypical lesions in the mandibular bone, highlighting the disease as an important differential diagnosis in similar presentations. These cases emphasize the ability of actinomycosis to mimic other conditions, underlining the importance of exhaustive differential diagnosis and the use of histological techniques for confirmation.

In 2006, Takasaki et al. [17] presented a case involving a 78-year-old woman with a significant unilateral enlargement of the tonsils caused by actinomycosis. The diagnosis was challenging, mainly due to difficulties in culturing the anaerobic organism. However, it was finally confirmed by the sulfur granules found in the biopsy sample. Treatment included tonsillectomy and prolonged antibiotic therapy, resulting in a favorable outcome. This case serves as a reminder of the rare ability of actinomycosis to cause significant unilateral enlargement of the tonsils and the importance of considering it in differential diagnoses.

In 2010, Rašić et al. [16] reported the case of a 14-year-old girl experiencing difficulty breathing, swallowing, and recurrent sore throat for 2 years. Unilateral tonsillar hypertrophy was observed, with actinomycosis confirmed through histopathological examination after tonsillectomy, revealing sulfur granules. Postoperative treatment with penicillin G led to a normal recovery, with no recurrence at 6 months, emphasizing actinomycosis as a differential diagnosis in similar presentations.

In 2008, Samant et al. [18] described a case of a 78-year-old diabetic woman presenting with severe sore throat and difficulty swallowing, initially misdiagnosed as a neoplasia. The histological examination after tonsillectomy revealed actinomycosis and a 6-week course of penicillin resulted in a complete recovery. This case highlights the potential of this disease to mimic neoplastic conditions and the crucial role of histological examination in accurate diagnosis.

In 2002, Yadav et al. [19] described the case of a 12-year-old girl with a massive unilateral enlargement of the tonsils initially suspected of being a tumor. The diagnosis of actinomycosis was established based on the results of post-surgery histopathological examination of the tonsils, with sulfur granules indicating infection. A 6-week course of penicillin therapy led to a complete recovery, underlining the need to consider actinomycosis in cases of atypical tonsil enlargement.

In 2014, Bulut et al. [20] reported a case of a 16-year-old patient who presented with a painless growing mass on the left cheek, with no history of trauma, but with scars and missing teeth noted during the oral examination. The diagnosis of *Actinomyces* spp. infection was based on findings of active chronic inflammation, abscess formation, and osteomyelitis, including the presence of sulfur granules and filamentous bacilli on histopathology. This case emphasizes the importance of differential diagnosis and appropriate treatment for atypical lesions of the mandibular bone.

In Table 3, a detailed comparison of clinical cases of actinomycosis is observed. It can be seen that the clinical manifestations of actinomycosis vary significantly according to the age and gender of the patients, as well as the location of the infection. For example, the case by Hasan and Kumar in 2011 [21] describes a 10-year-old girl with submandibular pain and difficulty swallowing, while Khatib et al. in 2015 [22] report a 42-year-old man with pain during swallowing for two months. These variations underscore the ability of actinomycosis to present symptoms that can mimic other conditions, complicating the diagnosis.

The procedures performed for the diagnosis and treatment of actinomycosis in the cases presented in the table predominantly include tonsillectomy. This procedure is common due to the frequent presentation of actinomycosis in the tonsils, as observed in the studies by Hasan and Kumar 2011 [21], Khatib et al. 2015 [22], Carvalho et al. 2016 [23], and Bold et al. 2013 [24]. Tonsillectomy allows for the collection of samples for histopathological examination, which is crucial for confirming the diagnosis of actinomycosis. Histopathological findings in all cases include the presence of sulfur granules, characteristic of actinomycosis. For example, Hasan and Kumar 2011 [21] observed neutrophilic infiltration and bacterial colonies with sulfur granules, while Carvalho et al. 2016 [23] identified nonspecific reactive follicular lymphoid hyperplasia and detected *Actinomyces* spp. using hematoxylin and eosin stain. These findings are essential for an accurate diagnosis and differentiate actinomycosis from other chronic granulomatous infections.

The treatment of actinomycosis generally involves the use of high-dose beta-lactam antibiotics for prolonged periods. For instance, Hasan and Kumar 2011 [21], used intravenous penicillin G followed by oral amoxicillin/clavulanate, while Bold et al. 2013 [24] employed roxithromycin for 14 days. The choice of antibiotic and the duration of treatment depend on the severity of the infection and the patient’s response to the initial treatment. 

Timely diagnosis of actinomycosis often faces challenges and may be deferred for months. This is due to the infection’s tendency to mimic malignant tumors, tuberculosis, or nocardiosis [1,4]. Thus, this report aims to raise awareness among health professionals about the importance of considering unusual differential diagnoses in complex clinical cases. *Actinomyces* spp. often show notable susceptibility to beta-lactams, particularly penicillin and amoxicillin, and with appropriate treatment over extended periods, generally between 6 and 12 months, complete eradication of the infection can be achieved [25,26,27,28].

Patients with actinomycosis require high doses and prolonged courses of antimicrobial therapy with beta-lactam antibiotics, and penicillin G, cephalosporin, or amoxicillin are frequently used. It is recommended to administer a dose of 18–24 million units per day of penicillin G intravenously for 2–6 weeks, followed by oral therapy with penicillin V or amoxicillin for 6–12 months [29]. However, other authors recommend that in certain regions, particularly the oral–cervicofacial region, the condition can be cured with a shorter duration of therapy, and treatment can be initiated with an oral agent. Generally, actinomycosis responds well to medical therapy. However, refractory or perceived refractory disease has been described in HIV-infected individuals and ostensibly normal hosts. [13]. It is suggested that *Actinomyces* spp. appears to be susceptible to a wide range of beta-lactam agents and these should be considered as first-choice agents when combined with beta-lactamase inhibitors, suggesting that accurate identification and speciation may impact clinical outcomes [13].

The comparative analysis between the presented case and other documented cases of actinomycosis highlights the diversity in clinical presentations and outcomes of this infectious disease, regardless of the patient’s age or medical history. This report focuses on a middle-aged man with a complex cancer history, specifically rhabdomyosarcoma, presenting a wide range of symptoms. This comprehensive diagnostic approach contrasts with previous cases, where patients of various ages displayed localized symptoms and were primarily diagnosed through histopathological examination after surgery. However, both this case and the previous ones confirm the effectiveness of antibiotic therapy, whether alone or in combination with surgery, for symptom resolution and achieving a favorable prognosis.

The uniqueness of this case lies in its specific clinical context, marked by the progression of actinomycosis in a patient with a cancer history. This case underscores the challenge of incorporating actinomycosis into the differential diagnosis, significantly differentiating it from other cases focused on oropharyngeal infections. Rare infections such as actinomycosis in patients with complex oncological histories must be considered, offering a unique perspective that contrasts with the focus on more typical disease presentations. This approach not only challenges usual differential diagnoses but also highlights the importance of a multidisciplinary approach in the diagnosis and treatment of actinomycosis, emphasizing the relevance of clinical vigilance and adaptability of therapeutic management in complex medical histories.

Moreover, the critical role of clinical surveillance in patients with significant medical histories is highlighted, where actinomycosis may not initially be considered because of the rarity of the disease and variable presentation. Another important issue is the universal effectiveness of antibiotic therapy, emphasizing the need for personalized approaches based on the clinical history and presentation of the patients. This joint analysis underscores the importance of an interdisciplinary approach in the diagnosis and management of actinomycosis, considering both typical and atypical presentations, and stresses the need for a high clinical suspicion for infectious diseases in patients with complex histories, including cancer, to ensure accurate diagnosis and provide effective treatments.

## 4. Conclusions

This case report underscores the critical importance of a multidisciplinary approach in the diagnosis and treatment of actinomycosis, a rare infectious disease that can mimic malignant conditions and common diseases such as tuberculosis. It emphasizes the need to consider unusual differential diagnoses in complex clinical scenarios, as demonstrated by the presented case of a patient with a complicated history of mandibular rhabdomyosarcoma. *Actinomyces* spp. infection was detected through the comprehensive use of advanced diagnostic techniques, despite the late consideration of this infection in the differential diagnosis. This case highlights the importance of maintaining a high clinical suspicion for atypical infections, particularly in complex clinical settings, and the variability in the clinical presentation of actinomycosis. The uniqueness of this case lies in the occurrence of actinomycosis in a patient with a history of cancer, highlighting the need to consider opportunistic infections in patients with chronic lesions and complex clinical pictures.

## Figures and Tables

**Table 1:** Patient admission laboratory values and reference ranges.

Test	Admission Value	Reference Range
Potassium (mEq/L)	4.35	3.5–5.1
Sodium (mEq/L)	142	132–146
Blood Urea Nitrogen (mg/dL)	14.1	9–23
Creatinine (mg/dL)	1.09	0.5–1.1
White Blood Cell Count (Absolute/µL)	8230	4050–11,840
Neutrophil Count (Absolute/µL)	5090	2070–7730
Hemoglobin (g/dL)	16.7	12.3–15.3
Platelet Count (µL)	276,000	203,000–445,000
Erythrocyte Sedimentation Rate (mm/h)	12	0–20
Alkaline Phosphatase	72	46–116
C—Reactive Protein (mg/dL)	0.2	0–10
AST (U/L)	23	0–34
ALT (U/L)	20	0–49

**Table 2:** Blood culture results.

Blood Cultures	Result
Fungal Blood Culture	Negative after 15 days of incubation
* Serial Blood Culture one	Negative after 5 days of incubation
* Serial Blood Culture one	Negative after 5 days of incubation

* Serial Blood Culture refers to a series of blood cultures taken at specific intervals to detect intermittent or persistent bacteremia or fungemia. This method increases the likelihood of identifying microorganisms that may not be present in a single blood sample [15].

**Table 3:** Comparative study of clinical presentation, procedures, histopathology, and treatment in actinomycosis cases.

Author	Age and Gender	Predominant Clinical Manifestation	Procedure Performed	Histopathology	Treatment
Hasan and Kumar, 2011 [21]	Female, Age: 10 years	Submandibular pain with painful swallowing	Elective tonsillectomy	Neutrophilic infiltration and bacterial colonies with sulfur granules confirming the presence of actinomycosis.	Intravenous penicillin G, 5 million units per day for 1 month, followed by oral penicillin (amoxicillin 400 mg + clavulanate 57 mg), twice a day for 2 months along with oral lactobacillus.
Khatib et al., 2015 [22]	Male, Age: 42 years	Pain during swallowing for the past 2 months	Bilateral tonsillectomy	Chronic tonsillitis with Actinomyces colonies. Periodic acid-Schiff staining confirmed the presence of Actinomyces.	Not specified
Carvalho et al., 2016 [23]	Female, Age: 63 years	Asymptomatic	Elective tonsillectomy	Nonspecific reactive follicular lymphoid hyperplasia and *Actinomyces* spp. detected. Hematoxylin and eosin stain highlights a bacterial cluster of *Actinomyces* spp. (“sulfur granule”).	Treatment with amoxicillin/clavulanic acid 500/125 mg, duration not specified.
Bold et al., 2013 [24]	Male, Age: 55 years	Two weeks of sore throat, dysphagia, odynophagia, cervical lymphadenopathy, fever, and severe asthenia	Tonsillectomy	Actinomyces confirmed by PAS and Giemsa stains.	Rovamycin for actinomycosis for 14 days.

## Data Availability

Data are contained within the article.

## Abbreviations

IUD	Intrauterine Device
GI	Gastrointestinal
CBC	Complete Blood Count
ARDS	Acute Respiratory Distress Syndrome
CT	Computed Tomography
MRI	Magnetic Resonance Imaging
VATS	Video-Assisted Thoracoscopic Surgery
ALP	Alkaline Phosphate
ERS	Erythrocyte Sedimentation Rate
TB	Tuberculosis
PET-CT	Positron Emission Tomography-Computed
